# Taxonomy and Identification of Principal Foliar Nematode Species (*Aphelenchoides* and *Litylenchus*)

**DOI:** 10.3390/plants9111490

**Published:** 2020-11-04

**Authors:** Zafar Handoo, Mihail Kantor, Lynn Carta

**Affiliations:** Mycology and Nematology Genetic Diversity and Biology Laboratory, USDA, ARS, Northeast Area, Beltsville, MD 20705, USA; mihail.kantor@usda.gov (M.K.); lynn.carta@usda.gov (L.C.)

**Keywords:** foliar nematodes, taxonomy, *Aphelenchoides*, *Litylenchus*

## Abstract

Nematodes are Earth’s most numerous multicellular animals and include species that feed on bacteria, fungi, plants, insects, and animals. Foliar nematodes are mostly pathogens of ornamental crops in greenhouses, nurseries, forest trees, and field crops. Nematode identification has traditionally relied on morphological and anatomical characters using light microscopy and, in some cases, scanning electron microscopy (SEM). This review focuses on morphometrical and brief molecular details and key characteristics of some of the most widely distributed and economically important foliar nematodes that can aid in their identification. *Aphelenchoides* genus includes some of the most widely distributed nematodes that can cause crop damages and losses to agricultural, horticultural, and forestry crops. Morphological details of the most common species of *Aphelenchoides* (*A. besseyi*, *A. bicaudatus*, *A. fragariae*, *A. ritzemabosi*) are given with brief molecular details, including distribution, identification, conclusion, and future directions, as well as an updated list of the nominal species with its synonyms. *Litylenchus* is a relatively new genus described in 2011 and includes two species and one subspecies. Species included in the *Litylenchus* are important emerging foliar pathogens parasitizing trees and bushes, especially beech trees in the United States of America. Brief morphological details of all *Litylenchus* species are provided.

## 1. Introduction

Foliar nematodes are mostly pathogens of ornamental crops in greenhouses, nurseries, and forest trees, as well as field crops [1]. Foliar nematodes include several nematode genera among which *Aphelenchoides*, *Anguina*, *Ditylenchus,* and *Litylenchus*. Foliar nematodes have been documented as associated with more than 1100 different species of plants, belonging to 126 botanical families, to include dicots, monocots, gymnosperms and angiosperms, ferns and mosses [2]. *Aphelenchoides,* as well as nematodes of genus *Litylenchus*, are phytoparasites known to infect leaves, stems, and buds [3]. The damage caused by the foliar nematodes can cause marketability problems in ornamentals because they interfere with the appearance of the plant or they can reduce yield in food crops [2]. 

## 2. General Techniques

For morphological observation, adult specimens of foliar nematodes can be extracted from fresh leaves. The best method for extracting nematodes from fresh leaves is by using the Baermann Funnel method. Another simple extraction method of nematodes from rice seeds was described by Hoshino and Togashi [4]. They cut the rice seeds longitudinally in two, then transferred the pieces into single plastic pipette tips, which were placed upright in glass vials with water. The pipet tips are transferred to new vials 2, 4, 8, and 24 h later the rate of nematode extraction can be observed. Nematodes are transferred to Syracuse watch glasses and counted. The final step includes dissection of seeds and counting the remaining nematodes after additional 24 h.

For light microscopic observation, fresh specimens are fixed using different methods, such as the ones described by Golden [5] and Hooper [6]. Another method of fixing nematodes was described by Ryss et al. [7] in which nematodes are placed into cold 4% formalin and 1% glutaraldehyde in 0.01 M phosphate buffer at pH 7.3, and then stored at 48 °C for light and scanning electron microscopy (SEM). 

For SEM observations, nematode specimens can be fixed in phosphate-buffered aldehyde and transferred to special chambers [8], rinsed for 15 min in distilled water, transferred for 2 h in 1% aqueous osmium tetroxide, rinsed again in distilled water and dehydrated in increasing concentrations of ethanol (10% to 100%) in 10% increments for 30 min each, followed by three changes of 100% ethanol. Alcohol is removed using a critical point dryer and the dried specimens stored under vacuum over silica gel. Dried specimens can be mounted on double-sided adhesive tape placed on SEM stubs, sputter-coated with 30 nm of gold, and photographed [7]. To observe the nematode stylets, one individual (alive) specimen is placed in a 1 µL drop of 45% lactic acid on a 12-mm-round, glass cover slip. A small sliver of a broken cover slip, approximately 1 × 3 mm, is placed over the specimen and pressure is applied to it with a needle until the nematode ruptures and the stylet and guiding apparatus extrude. After 24 h, small triangles of filter paper are applied to the edge of the broken sliver to remove the lactic acid, which is exchanged with 2% formalin, followed by three changes of 50% ethanol. The sliver is then floated by adding 50% ethanol and removed with forceps. Stylets adhering to the glass cover slips are air-dried and prepared for SEM as described previously.

Other methods to prepare nematodes for low temperature SEM observations were described by Kantor et al. [9] and Carta et al. [10]. Nematodes can be placed in 1.5 Eppendorf tubes filled with a fixative composed of 2% Paraformaldehyde, 2.5% Glutaraldehyde, 0.05 M Na Cacodylate, and 0.005 M CaCl_2_; for at least 12 h. After 12 h, specimens are rinsed in distilled water and individual nematodes placed onto ultra-smooth, round (12 mm diameter), carbon adhesive tabs (Electron Microscopy Sciences, Inc., Hatfield, PA, USA) secured to 15 mm × 30 mm copper plates. The nematode specimens are frozen conductively, in a Styrofoam box, by placing the plates on the surface of a pre-cooled (−196 °C) brass bar whose lower half is submerged in liquid nitrogen. After 20–30 s, the brass plate containing the frozen sample is transferred to the Quorum PP2000 cryo transfer system (Quorum Technologies, East Sussex, UK), attached in this case to an S-4700 field emission scanning electron microscope (Hitachi High Technologies America, Inc., Dallas, TX, USA). The specimens are freeze- etched inside the cryotransfer system to remove any surface contamination (condensed water vapor) by raising the temperature of the stage to −90 °C for 10–15 min. Following etching, the temperature inside the chamber is lowered below −130 °C, and the specimens coated with a 10 nm layer of platinum using a magnetron sputter head equipped with a platinum target. The specimens are transferred to a pre-cooled (−130 °C) cryostage in the SEM for observation. An accelerating voltage of 5 kV is used to view the specimens. 

DNA extraction from live specimens can be performed using the freeze-thaw lysis with a single live nematode in a 0.2 mL PCR tube containing 25 μL of extraction buffer (10 mM Tris pH 8.2, 2.5 mM MgCl_2_, 50 mM KCl, 0.45% TWEEN 20 and 0.05% gelatin). Next, the PCR tube is submerged in liquid nitrogen for 10 to 15 s and then placed at 95 °C for 2 min in a thermal cycler. The tube is submerged one more time in liquid nitrogen for 10 to 15 sec and then slow-thawed at room temperature. After thawing, the sample is lysed with 1 µL of proteinase K (800 U/mL, Sigma-Aldrich, St. Louis, MO, USA) at 60 °C for 60 min, followed by 95 °C for 15 min to deactivate the proteinase K. It is recommended to use at least three single nematodes for the individual DNA extraction. The lysates can be stored at −20 °C until needed [11,12]. After extraction, the DNA fragments can be amplified using SSU rDNA (18S), D2D3 (28S) expansion region of the LSU rDNA and cytochrome oxidase subunit 1 of the mitochondrial DNA (mtCOI) markers [13]. The primers used for the 18S fragment amplification are 1813F (CTGCGTGAGAGGTGAAAT) and 2646R (GCTACCTTGTTACGACTTTT) and were first published by Holterman et al. [14]. Primers used for the amplification of the 28S region are D2A (ACAAGTACCGTGAGGGAAAGTTG) and D3B (TCCTCGGAAGGAACCAGCTACTA) [15]. The mtCOI fragment can be amplified using COI-F1(CCTACTATGATTGGTGGTTTTGGTAA TTG) and COI-R2 (GTAGCAGCAGTAAA ATAAGCACG) primers [16]. 

## 3. Genus *Aphelenchoides* Fischer, 1894

After Goodey [17] the genus *Aphelenchoides* Fischer, 1894 is characterized as follows:

- Six fused, non-annulated, similar lips, slightly offset from body;
- Male tail without bursa, with one pair of approximately adanal and two pairs of postanal, ventro-submedian, caudal papillae;
- Spicules paired and shaped like rose thorns;
- Tails of both sexes never elongate filiform but short, tapering, conical, and frequently ending in one or more mucrones.

A polytomous key was developed and tested on 14 populations by Hockland [18] and the primary key characters were identified as:

- The length of the post-vulval sac;
- The shape of the tail terminus and tail;
- Body length;
- Ratios ‘a’ and ‘c’.

A more detailed characterization of the genus was first given by Allen [19]:

- Cuticle marked by fine transverse striae;
- Lateral field marked as longitudinal incisures;
- Lip region set off from body;
- Six lips supported by six radial internal sclerotization;
- Lips not annulated;
- Stylet with or without basal knobs;
- Medial esophageal bulb well developed;
- Intestine joining esophagus immediately behind bulb;
- Nerve ring encircling anterior ends of intestine and the esophageal glands;
- Esophageal glands free in the body cavity;
- Single anteriorly directed ovary, oocytes on tandem or multiple;
- Male tail without bursa or gubernaculum;
- Three pairs of ventro-submedian papillae usually present on male tail;
- Spicules paired, ventrally arcuate.

Female and male tail never elongate filiform. A recent characterization of *Aphelenchoides* was given by Wheeler and Crow [20]:

- Stylet with small basal knobs;
- Males are common;
- Vulva located near 2/3 the body length from the anterior;
- Prodelphic (anteriorly outstretched) ovary and a post-uterine sac;
- Males have prominent, thorn-shaped spicules (paired, cuticularized copulatory structures).

According to Hunt [21] members of the *Aphelenchoides* genus can be diagnosed by the following morphological characteristics:

- Body length between 0.4 to 1.2 mm (commonly from 0.4 to 0.8 mm);
- Females become straight to ventrally arcuate when heat relaxed while males assume a “walking-stick shape”;
- Cuticle finely annulated, two to four (rarely six) incisures in the lateral field;
- Stylet slender with basal knobs (sometimes indistinct), length between 10–12 μm;
- Long and slender procorpus; well-developed spherical to rounded-rectangular shaped metacorpus, with central valve plates; esophageal gland lobe long, with dorsal overlap of the intestine;
- Vulva usually between 60 and 75% of the body length;
- Ovary monoprodelphic, typically outstretched, but may reflex;
- Post-vulval sac present most of the times;
- Oocytes in one or more rows;
- Post-uterine sac present (sometimes absent) and most of the times contains spermatozoa;
- Tail shape is conoid to variable; males have a tail more strongly curved ventrally and papillae variable;
- Tail terminus with one or more mucros or without mucros;
- Spicules well-developed, thorn-shaped, paired and separate without bursa.

## 4. Genus *Aphelenchoides* Fischer, 1894

Emended Diagnosis [22]

These nematodes are small and slender, averaging around one millimeter in length and a width less than 20 microns. One characteristic of thee Aphelenchidae nematodes family is that they have a larger median bulb as compared to other plant parasitic nematodes in the order Tylenchida. The dorsal esophageal gland orifice connects to the esophageal lumen at the base of the stylet in most plant-parasitic nematodes, but in Aphelenchida, this duct empties into the esophageal lumen within the median bulb. In *Aphelenchoides*, males are more common, and they reproduce primarily by amphimixis. In most species, the vulva of the female is located near 2/3 the body length from the anterior. Females have a single, prodelphic (anteriorly outstretched) ovary and a post-uterine sac, while males have prominent, thorn-shaped spicules (paired, cuticularized copulatory structures). There is a considerable variation in the shape of the tail terminus within populations of species of the genus *Aphelenchoides.* The tail terminus can be used to divide *Aphelenchoides* species into four groups [22]. The four groups are:

- Tail without any outgrowth or mucro;
- Tail with one or sometimes two mucronate structures on tail end;
- Star shaped tail with four mucronate structures;
- Tail end with outgrowth other than spine or star.

## 5. Systematic Position

The number of valid nominal species in the Aphelenchoidea is still debatable. However, modern molecular technology may help solve this problem soon. According to Hunt [23] there are 453 ‘valid’ species in Aphelenchoidea, of which 33 belong to the Aphelenchidae and 420 to the Aphelenchoididae. From Aphelenchoididae family, *Aphelenchoides* genera has the most species, namely 153 [23]. A more recent 2015 study conducted by Sánchez-Monge et al. [2] assigned approximately 200 species to the genus. However, after conducting a through literature review, the authors have identified 182 valid nominal species assigned to the *Aphelenchoides* genus. 

## 6. Diagnostic Characters

Some diagnostic characteristics of *Aphelenchoides* are presented below [3]:

- Slender body, length variable;
- Lips often slightly offset;
- Stylet with basal knobs;
- Oocytes in one or more rows;
- Post-uterine sac usually well-developed, with variable length;
- Spicules paired, rose thorn-shaped, not fused, rostrum usually prominent;
- Male tail without caudal alae or gubernaculum; with three pairs of ventro-submedian papillae;
- Tails of both sexes never elongate-filiform, but usually more or less tapering, conical, and frequently ending in one or more mucrons.

## 7. Genus Synonyms

Emended list of *Aphelenchoides* species and synonyms:

**Type species:**

1. *A. kuehnii* Fischer, 1894 = *A.* (*Aphelenchoides*) *kuehnii* Fischer, 1894 (Filipjev, 1934)

**Other species:**

1. *A. absari* Husain and Khan, 1967
2. *A. abyssinicus* (Filipjev, 1931) Filipjev, 1934 = *Aphelenchus abyssinicus* Filipjev, 1931
3. *A. aerialis* Chanu, Mohilal, Victoria and Shah, 2015
4. *A. africanus* Dassonville and Heyns, 1984
5. *A. agarici* Seth and Sharma, 1986
6. *A. aligahriensis* Siddiqi, Hussain and Khan, 1967
7. *A. andrassyi* Husain and Khan, 1967
8. *A. angusticaudatus* Eroshenko, 1968
9. *A. appendurus* Singh, 1967
10. *A. arachidis* = *Robustodorus arachidis* Bos, 1977
11. *A. arcticus* Sanwal, 1965
12. *A. asterocaudatus* Das, 1960
13. *A. asteromucronatus* Eroshenko, 1967
14. *A. baguei* Maslen, 1979
15. *A. besseyi* Christie, 1942 = *Aphelenchoides oryzae* Yokoo, 1948 *Asteroaphelenchoides besseyi* (Christie 1942) Drozdovski, 1967
16. *A. bicaudatus* (Imamura, 1931) Filipjev and Schuurmans Stekhoven, 1941 = *Aphelenchus bicaudatus* (Imamura, 1931)
17. *A. bimucronatus* Nesterov, 1985
18. *A. blastophthorus* Franklin, 1952
19. *A. brassicae* Edward and Misra, 1969
20. *A. brevicaudatus* Das, 1960
21. *A. brevionchus* Das, 1960
22. *A. breviuteralis* Eroshenko, 1967
23. *A. brushimucronatusBajaj* and *Walia*, 1999
24. *A. capsuloplanus* = *Paraphelenchoides capsuloplanus* Haque, 1967
25. *A. centralis* Thorne and Malek, 1968
26. *A. chalonus* Chawla and Khan, 1979
27. *A. chamelocephalus* (Steiner, 1926) Filipjev, 1934
28. *A. chauhani* Tandon and Singh, 1974
29. *A. chinensis* Husain and Khan, 1967
30. *A. cibolensis* Riffle, 2011
31. *A. citri* Andrássy, 1957
32. *A. clarolineatus* Baranovskaya, 1958
33. *A. clarus* Thorne and Malek, 1968
34. *A. composticola* Franklin, 1957
35. *A. confusus* Thorne and Malek, 1968
36. *A. conimucronatus* Bessarabova, 1966
37. *A. conophthori* Massey, 1974
38. *A. curiolis* Gritsenko, 1971
39. *A. cyrtus* Paesler, 1957
40. *A. dactylocercus* Hooper, 1958
41. *A. dalianensis* Cheng, Hou and Lin, 2009
42. *A. daubichaensis* Eroshenko, 1968
43. *A. delhiensis* Cwala, Bhamburkar, Khan and Prasad, 1968
44. *A. dhanachandhi* Chanu, Mohilal and Shaw, 2012
45. *A. dubitus* Ebsary, 1991
46. *A. echinocaudatus* Haque, 1968
47. *A. eldaricus* Esmaeili, Heydari, Golhasan and Kanzaki, 2017
48. *A. editocaputis* Shavrov, 1967
49. *A. eltayebi* Zeidan and Geraert, 1991
50. *A. emiliae* Romaniko, 1966
51. *A. ensete* Swart, Bogale and Tiedt, 2000
52. *A. eradicitus* Eroshenko, 1968
53. *A. fluviatilis* Andrassy, 1960
54. *A. fragariae* (Ritzema Bos, 1891) Christie, 1932 = *Aphelenchoides olesistus* (Ritzema Bos, 1893) Steiner, 1932 *Aphelenchoides olesistus* var. *longicollis* (Schwartz, 1911) Goodey, 1933 *Aphelenchoides pseudolesistus* (Goodey, 1928) Goodey, 1933 *Aphelenchus fragariae* Ritzema Bos, 1891 *Aphelenchus olesistus* Ritzema Bos, 1893 *Aphelenchus olesistus* var. *longicollis* Schwartz, 1911 *Aphelenchus pseudolesistus* Goodey, 1928
55. *A. franklini* Singh, 1969
56. *A. fuchsi* Esmaeili, Heydari, Ziaie and Gu, 2016
57. *A. fujianensis* Zhuo, Cui, Ye, Luo, Wang, Hu, and Liao, 2010
58. *A. giblindavisi* Aliramaji, Pourjam, Alvarez-Ortega, Afshar and Pedram, 2017
59. *A. goeldii* (Steiner, 1914) Filipjev, 1934 = *Aphelenchus goeldii* Steiner, 1914 *Aphelenchoides* (A.) *goeldii* (Steiner, 1914) Filipjev, 1934
60. *A. goldeni* Suryawanshi, 1971
61. *A. goodeyi* Siddiqi and Franklin, 1967
62. *A. gorganensis* Miraeiz, Heydari and Bert, 2017
63. *A. graminis* Baranovskaya and Haque, 1968
64. *A. gynotylurus* Timm and Franklin, 1969
65. *A. haguei* Maslen, 1978
66. *A. hamatus* Thorne and Malek, 1968
67. *A. heidelbergi* Carta, Li, Skantar, and Newcombe, 2016 = *Laimaphelenchus heidelbergi* Zhao, Davies, Riley, and Nobbs, 2007
68. *A. helicosoma* Maslen, 1978
69. *A. helicus* Heyns, 1964
70. *A. helophilus* (de Man, 1880) Goodey, 1933 = *Aphelenchus helophilus* le Man, 1880 *Aparietinus var. helophilus* de Man, 1880 *Aphelenchoides (A.) helophilus* (de Man, 1880) Goodey, 1933 *Aphelenchus elegans* Micoletzky, 1913
71. *A. heterophallus* Steiner, 1934
72. *A. huntensis* Esmaeili, Fang, Li and Heydari, 2016
73. *A. hunti* Steiner, 1935
74. *A. hylurgi* Massey, 1974
75. *A. indicus* Chawla, Bhamburkar, Khan and Prasad, 1968
76. *A. involutus* Minegawa, 1992
77. *A. iranicus* Golhasan, Heydari, Alvarez-Ortega and Palomares-Rius, 2016
78. *A. jacobi* Husain and Khan, 1967
79. *A. jodhpurensis* Tikyani, Khera and Bhatnagar, 1970
80. *A. jonesi* Singh, 1977
81. *A. kheirii* Golhasan, Heydari, Esmaeili and Kanzaki, 2018
82. *A. kungradensis* Karimova, 1957
83. *A. lanceolatus* Tandon and Singh, 1974
84. *A. lagenoferrus* Baranovskaya, 1963
85. *A. lanceolatus* Tandon and Singh, 1974
86. *A. lichenicola* Siddiqi and Hawksworth, 1982
87. *A. lilium* Yokoo, 1964
88. *A. limberi* Steiner, 1936 = *Paraphelenchoides limberi* (Steiner, 1936) Hague, 1967
89. *A. longiurus* Das, 1960
90. *A. longiuteralis* Eroshenko, 1967
91. *A. loofi* Kumar, 1982
92. *A. lucknowensis* Tandon and Singh, 1973
93. *A. macromucrons* Slankis, 1967
94. *A. macronucleatus* Baranovskaya, 1963
95. *A. macrospica* Golhasan, Heydari, Esmaeili and Miraeiz, 2017
96. *A. marinus* Timm and Franklin, 1969
97. *A. martinii* Ruhm, 1955
98. *A. medicagus* Wang, Bert, Gu, Couvrer and Li, 2019
99. *A. meghalayensis* Bina and Mohilal, 2017
100. *A. menthae* Lisetzkaya, 1971
101. *A. microsylus* Kaisa, 2000
102. *A. minor* Seth and Sharma, 1986
103. *A. myceliophagus* Seth and Sharma, 1986
104. *A. nechaleos* Hooper and Ibrahim, 1994
105. *A. neocomposticola* Seth and Sharma, 1986
106. *A. neoechinocaudatus* Chanu, Mohilal and Shah, 2012
107. *A. nonveilleri* Andrassy, 1959
108. *A. obtusicaudatus* Eroshenko, 1967
109. *A. obtusus* Thorne and Malek, 1968
110. *A. orientalis* Eroshenko, 1968
111. *A. pannocaudus* Massey, 1966
112. *A. paradalianensis* Cui, Zhuo, Wang and Liao, 2011
113. *A. paramonovi* Eroshenko and Kruglik, 2004
114. *A. paranechaleos* Hooper and Ibrahim, 1994
115. *A. parasaprophilus* Sanwal, 1965
116. *A. parasexalineatus* Kalinich, 1984
117. *A. montanus* Singh, 1967
118. *A. panaxi* Skarbilovich and Potekhina, 1959
119. *A. parabicaudatus*, Shavrov, 1967
120. *A. parascalacaudatus* Chawla, Bhamburkar, Khan and Prasad, 1968
121. *A. parasubtenuis* Shavrov, 1967
122. *A. paraxui* Esmaeili, Heydari, Fang and Li, 2017
123. *A. parietinus* (Bastian, 1865) Steiner, 1932
124. *A. petersi* Tandon and Singh, 1970
125. *A. pinusi* Bajaj and Walia, 1999
126. *A. pityokteini* Massey, 1974
127. *A. platycephalus* Eroshenko, 1968
128. *A. polygraphi* Massey, 1974
129. *A. primadentus* Esmaeili, Heydari, Golhasan and Kanzaki, 2018
130. *A. pseudogoodeyi* Oliveira, Subbotin, Alvarez-Ortega, Desaeger, Brito, Xavier, Freitas, Vau and Inserra, 2019
131. *A. pusillus* (Thorne, 1929) Filipjev, 1934
132. *A. rarus* Eroshenko, 1968
133. *A. rhytium* Massey, 1971
134. *Aphelenchoides ritzemabosi* (Schwartz, 1911) Steiner and Buhrer = *Aphelenchoides ribes* (Taylor, 1917) Goodey, 1933; *Aphelenchus phyllophagus* Stewart, 1921; *Aphelenchus ribes* (Taylor, 1917) Goodey, 1923; *Aphelenchus ritzemabosi* (Schwartz, 1911); *Pathoaphelenchus ritzemabosi* (Schwartz, 1911) Steiner, 1932; *Pseudaphelenchoides ritzemabosi* (Schwartz, 1911) Drozdovski, 1967; *Tylenchus ribes* Taylor, 1917
135. *A. rosei* Dmitrenko, 1966
136. *A. rotundicaudatus* Fang, Wang, Gu and Li, 2014
137. *A. rutgersi* Hooper and Myers, 1971
138. *A. sacchari* Hooper, 1958
139. *A. sanwali* Chaturvedi and Khera, 1979
140. *A. saprophilus* Franklin, 1957
141. *A. salixae* Esmaeili, Heydari, Tahmoures and Ye, 2017
142. *A. scalacaudatus* Sudakova, 1958
143. *A. seiachicus* Nesterov, 1973
144. *A. sexlineatus* Eroshenko, 1967
145. *A. shamimi* Khera, 1970
146. *A. siddiqii* Fortuner, 1970
147. *A. silvester* Andrassy, 1968
148. *A. sinensis* (Wu and Hoeppli, 1929) Andrassy, 1960
149. *A. singhi* Das, 1960
150. *A. sinodendroni* Ruhn, 1957
151. *A. smolae* Cai, Gu, Wang, Fang and Li, 2020
152. *A. solani* Steiner, 1935
153. *A. spasskii* Eroshenko, 1968
154. *A. sphaerocephalus* Goodey, 1953
155. *A. spicomucronatus* Truskova, 1973
156. *A. spinosus* Paesler, 1957
157. *A. spinohamatus Bajaj* and *Walia*, 1999
158. *A. spinosus Paesler*, 1957
159. *A. stammeti Korner*, 1954
160. *A. steineri Ruhm*, 1956
161. *A. stellatus* Fang, Gu, Wang and Li, 2014
162. *A. submersus* Truskova, 1973
163. *A. subparietinus Sanwal,* 1961
164. *A. subtenuis* = *Robustodorus subtenuis* (Cobb, 1926) Steiner and Buhrer, 1932
165. *A. suipingensis* Feng and Li, 1986
166. *A. swarupi* Seth and Sharma, 1986
167. *A. tabarestanensis* Golhasan, Fang, Li, Maadi and Heydari, 2019
168. *A. tagetae* Steiner, 1941
169. *A. taraii* Edward and Misra, 1969
170. *A. tsalolikhini* Ryss, 1993
171. *A. trivialis* Franklin and Siddiqi, 1963
172. *A. tumulicaudatus* Truskova, 1973
173. *A. turnipi* Israr, Shahina and Nasira, 2017
174. *A. tuzeti* B’Chir, 1978
175. *A. unisexus* Jain and Singh, 1984
176. *A. varicaudatus* Ibrahim and Hooper, 1994
177. *A. vaughani* Maslen, 1978
178. *A. vigor* Thorne and Malek, 1968
179. *A. wallacei* Singh, 1977
180. *A. xui* Wang, Wang, Gu, Wang and Li, 2013
181. *A. zeravschanicus* Tulaganov, 1948

## 8. Principal Species

The following four species have been selected for further discussion because of their commonality, economic importance, and/or worldwide distribution:

- *Aphelenchoides besseyi* Christie, 1942;
- *Aphelenchoides bicaudatus* (Imamura, 1931) Filipjev and Schuurmans Stekhoven;
- *Aphelenchoides fragariae* (Ritzema Bos, 1891) Christie, 1932;
- *Aphelenchoides ritzemabosi* (Schwartz, 1911) Steiner and Buhrer, 1941.

Each species is illustrated below (Figures 1–11). Data were obtained from various sources, including Allen [19]; Christie [24] De Jesus et al. [25], 2016; Xu et al. [26]; Siddiqi [27,28,29,30]; Shahina [22]; Siddiqui and Taylor [31]; Jen et al. [32]; Khan et al. [33]; Chizhov et al. [34]; Zhao et al. [35]; Khan et al. [36]; Hunt [21], Kanzaki et al. [37] 2019 and Carta et al. [11], and original descriptions and/or re-descriptions.

Because *Aphelenchoides besseyi* Christie, 1942, *Aphelenchoides fragariae* (Ritzema Bos, 1891) Christie, 1932, *Aphelenchoides ritzemabosi* (Schwartz, 1911) Steiner and Buhrer*, Aphelenchoides bicaudatus* (Imamura, 1931) Filipjev and Schuurmans Stekhoven, 1941 are of major economic importance and widely distributed all over the world, they will be discussed in detail.

## 9. Rice White-Tip Nematode (*Aphelenchoides besseyi* Christie, 1942)

**Figure 1:**
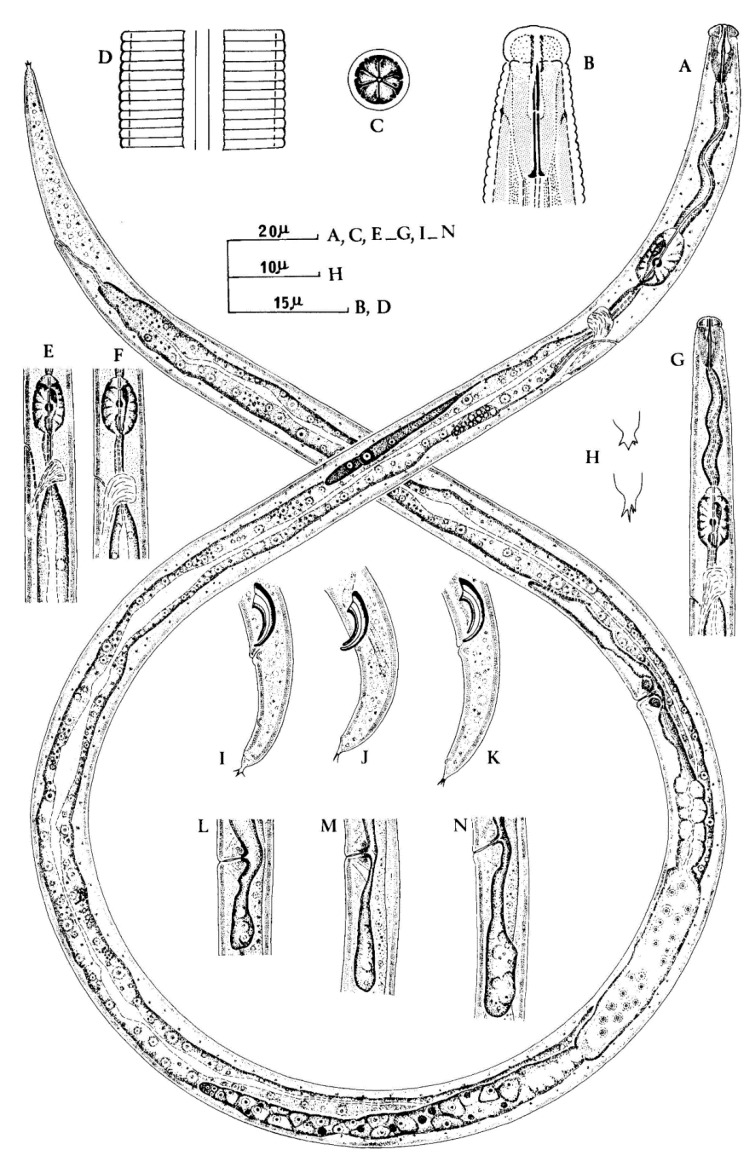
*Aphelenchoides besseyi* Christie (**A**) female; (**B**) female head end; (**C**) female en face view; (**D**) Lateral field; (**E**,**F**) variation in female esophageal bulb and position of excretory pore with respect to nerve ring; (**G**) male anterior end; (**H**) female tail termini showing variation in shape mucro; (**I**–**K**) male tail ends; (**L**–**N**) variation in post-vulval uterine sac (**B** and **D** original, the rest after Fortuner, 1970) after Franklin and Siddiqi [27]. Courtesy of *Commonwealth Institute of Helminthology*.

*Aphelenchoides besseyi* (Figure 1) is an economically important pathogen of rice and has been reported from many countries. However, it is not commonly found in ornamentals [38,39], with the exception of some reports on tuberose [36], begonia [40], gerbera [41], hydrangea [27], tuberose [42], and even on bird nest fern [43]. *A. besseyi* distribution is mostly in warmer climates, whereas *A. ritzemabosi* and *A. fragariae* are more commonly associated with temperate climates, while found in both tropical and temperate localities [1].

Measurements

After Christie [24].

Females (*n* = 10): length = 0.66–0.75 mm; a = 32–42 (width = 17–22); b = 10.2–11.4 (esophagus = 64–68 µm); c = 17–21 (tail = 36–42 µm); V = 68–70%.

Males (*n* = 10): length = 0.54–0.62 mm; a = 36–39 (width = 14–17 µm); b = 8.6–8.8 (esophagus = 63–66 µm); c = 15–17 (tail = 34–37 µm); T = 44–61% 

After Allen [19].

Females: length = 0.62–0.88 mm; a = 38–58; b = 9–12; c = 15–20; V = 66–72

Males: length = 0.44–0.72 mm; a = 36–47; b = 9–11; c = 14–19; T = 50–65%.

After De Jesus et al. [20]

Females: length = 0.65–0.75 mm; a = 42.8–49; c = 15.6–17.5; c’ = 4.0–4.5.

Males: length = 0.65–0.75 mm; a = 42.8–49; c = 15.6–17.5; c’ = 4.0–4.5; spicule = 14.1–18.3 µm.

After Xu et al. [44]

Body length (*n* = 11) = 0. 656 ± 18.5 (0.546–0.729) mm; body width = 14.4 ± 0.32 (12.4–15.9) µm; pharynx = 124 ± 2.53 (111.0–137.8) µm; stylet = 12.5 ± 0.21 (10.6–13.3) µm; median bulb end to anterior end 69.7 ± 1.07 (65.7–75.3) µm; tail length 36.9 ± 0.38 (35.3–38.9) µm; anus/cloacal width 9.27 ± 0.47(7.5–12.1) µm.

Description

**Figure 2:**
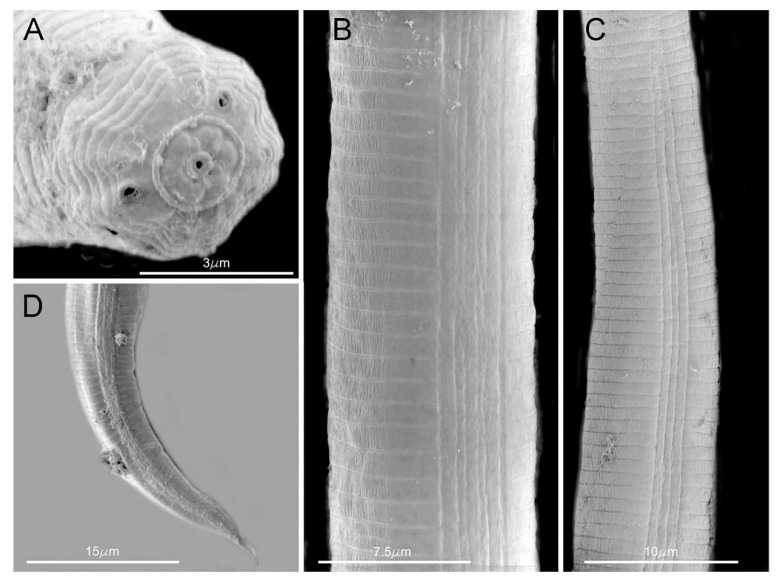
SEM photomicrographs of *Aphelenchoides besseyi* female (**A**) head end; (**B**,**C**) lateral fields; (**D**) tail end, after Khan et al. [36]. Courtesy of *Journal of Nematology*.

Female: female specimens share a slender body, slightly arcuate ventrally when relaxed, anteriorly tapering from the level of esophageal glands to the head, which is one half of the body width. Four lateral lines (occasionally six noted) are present in the lateral field (Figure 2). In en face view, the pore-like amphids are on outer margins of lateral lips; four papillae, one on each submedian lip (Figure 2). Lip region is non-striated and set off from body by a constriction as wide as or slightly wider than adjacent body; labial framework weakly developed; cheilorhabdions well sclerotized. Basal knobs of spear distinct, 2 µm across. Procorpus cylindrical; median esophageal bulb one and a half times to twice as long as wide, with refractive valvular apparatus slightly posterior to center. Esophageal glands extending over intestine 5 to 8 body widths. Excretory pore at 58 to 83 µm from anterior end, level with or slightly anterior to nerve ring. Hemizonid distinct in specimens from rice seeds (but not from cultured specimens), 11 to 15 µm behind excretory pore; hemizonion 20 to 30 µm behind hemizonid, usually difficult to see. Tail straight, slender, regularly tapering to a narrowly rounded end, 3–5 to 5 anal body diameters long; mucro with 3 to 4 processes. Ovary not extending to esophageal glands; oocytes in 2 to 4 rows; spermatheca very conspicuous, elongate oval, full of rounded sperms showing a central nucleolus usually surrounded by a circle of black dots of unknown nature. Post-vulval uterine sac short, slender and extending up to one fourth of the distance from vulva to anus (2.5 to 3 body diameters) often found empty and collapsed but more conspicuous and rounded in nematodes from cultured specimens. Vulval lips slightly protruding after Fortuner [45].

Male: tail end usually curved by 90° (a greater curvature has also been found) in specimens killed in 3% formaldehyde; mucro of diverse shape, with 2 to 4 processes. Spicule length between 17 to 21 µm along dorsal limb. Different morphometric characters, such as the shape of the head, the position of the excretory pore in relation to the nerve ring and the shape and length of the post-vulval uterine sac were found to be variable between populations [45]. 

Distribution

According to Devran et al. [46], *A. besseyi* was on the quarantine lists of nine countries in 1982 and up to 70 countries in 2002. Centre for Agriculture and Biosciences International (CABI), Invasive Species Compendium [47] lists *A. besseyi* being present in 75 countries around the world. The quarantine pests lists *A. besseyi* as the second most prevalent nematode after *Globodera rostochiensis* [39]. 

*Aphelenchoides bicaudatus* (Imamura, 1931) Filipjev and Schuurmans Stekhoven, 1941 *Aphelenchoides bicaudatus* (Imamura, 1931) Filipjev and Schuurmans Stekhoven, 1941 was originally described from a paddy field in Japan and previously considered a primarily mycophagous species. Since then, it has been reported to parasite more than 200 plant species [31,48].

Measurements

After Imamura [49].

Female (*n* = 18): L = 0.38–0.47 (0.43) mm; a = 31.3–31.7 (31.5); b = 6.8–8.4 (7.4); c = 9.4–12.6 (10.6); V%= 61.7–90.2 (0.4).

After Siddiqui and Taylor [31]. 

Female (*n* = 50): L = 0.41–0.55 (0.46) mm; a = 25–31 (28.0); b = 7.3–9.6 (8.2); c = 9.8–13.7 (11.4); V% = 65–70 (67.5); stylet = 10–12 (11.2) µm.

Male: L = 0.385 mm; a = 22.6; b = 7.5; c = 11.4; stylet = 10 µm.

After Jen et al. [32].

Female (*n* = 50): L = 499.12 ± 67.95 (0.376–0.637) mm; maximum body width = 15.24 ± 2.69 (11–22) µm; a = 33.03 ± 2.42 (27.00–38.64); b = 9.0 ± 0.7 (7.5–10.0); b’ = 5.13 ± 0.76 (3.61–7.94); c = 11.94 ± 0.93 (10.16–14.80); c’ = 5.41 ± 0.56 (4.13–7.14); V% = 68.53 ± 1.20 (64.90–71.83); stylet = 10.38 ± 0.63 (9–12) µm; length of post-uterine sac expressed as % of length from vulva to anus = 18.98 ± 4.54 (9.23–33.80) µm. 

After Israr et al. [50].

Female (*n* = 2): L = 0.36 mm; a = 30.1, 32.7; b = 8.8,7.2; b’ = 5.6, 5.8; c = 11.3, 12; c’ = 2.9, 3.7; V% = 66.8–67.2; G_1_% = 25, 26.2; body diameter 12, 12,5; stylet = 10, 11 µm, median bulb length 10, 10 µm; median bulb width 7, 8 µm; median bulb length/ width 1.4, 1.3; distance anterior end to distal end of median bulb 51, 52 µm; anterior end to excretory pore 50, 51 µm; anterior end to nerve ring 55, 56 µm; anterior end to vulva 242, 248 µm; ovary length 95, 84 µm; distance from vulva to anus 85, 84 µm; post uterine sac length 24, 22 µm; post uterine sac length/vulva anus distance% 22.4, 24; esophageal length 90, 92 µm; esophageal intestinal junction 62, 64 µm; tail length 31, 30 µm; anal body width 31, 30 µm, anal body width 11, 8. 

Male (*n* = 1): L = 0.40 mm; a = 30.7; b = 4.3; b’ = 6.2; c = 10; c’ = 3.9; T% = 52; body diameter 13; stylet = 10 µm, median bulb length 12 µm; median bulb width 9 µm; median bulb length/width 1.3; distance anterior end to distal end of median bulb 54 µm; anterior end to excretory pore 62 µm; anterior end to nerve ring 60 µm.

Description

**Figure 3:**
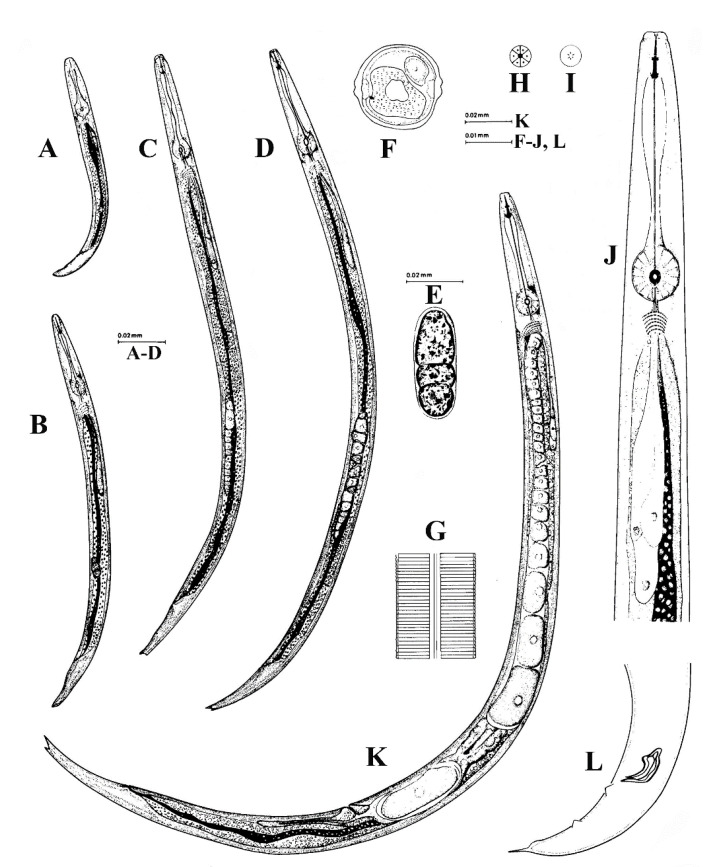
*Aphelenchoides bicaudatus* (Imamura) Filip. and Sch. Stek (**A**–**D**) Larvae, first of fourth stages; (**E**) egg; (**F**) cross section of female at mid-body; (**G**) lateral field; (**H**) face view; (**I**) framework around oral opening; (**J**) esophageal region in dorsal view; (**K**) whole female; (**L**) male tail after Siddiqi [28]. Courtesy of *Commonwealth Institute of Helminthology*.

Female: have a slender body, attenuated slightly anteriorly, and more prominently toward posterior end (Figure 3). When relaxed by gentle heat the position of the body is straight and only the tail region is slightly curved. Cuticle is finely striated, with annuli measuring between 0.47–0.58 μm wide and 0.39–0.51 μm thick. Lateral field has two lateral lines. Head distinctly set off from body. Lip region rounded, offset with no annules. Stylet weak, with small basal swellings. Metacorpus rounded, occupying approximately 73% of body width. Nerve ring is located about 1/2 body width behind metacorpus. Excretory pore opposite anterior margin of nerve ring. Vulva a transverse slit and slightly protruding, about 66% of body length from anterior end. Post-vulvar uterine sac extending for one-fifth of distance from vulva to end of tail. Rectum prominent, straight, near ventral body wall, and in length approximately three-fourths of anal body width. Tail gradually tapering to terminus, which is unevenly bifurcated with one prong longer than the other. 

**Figure 4:**
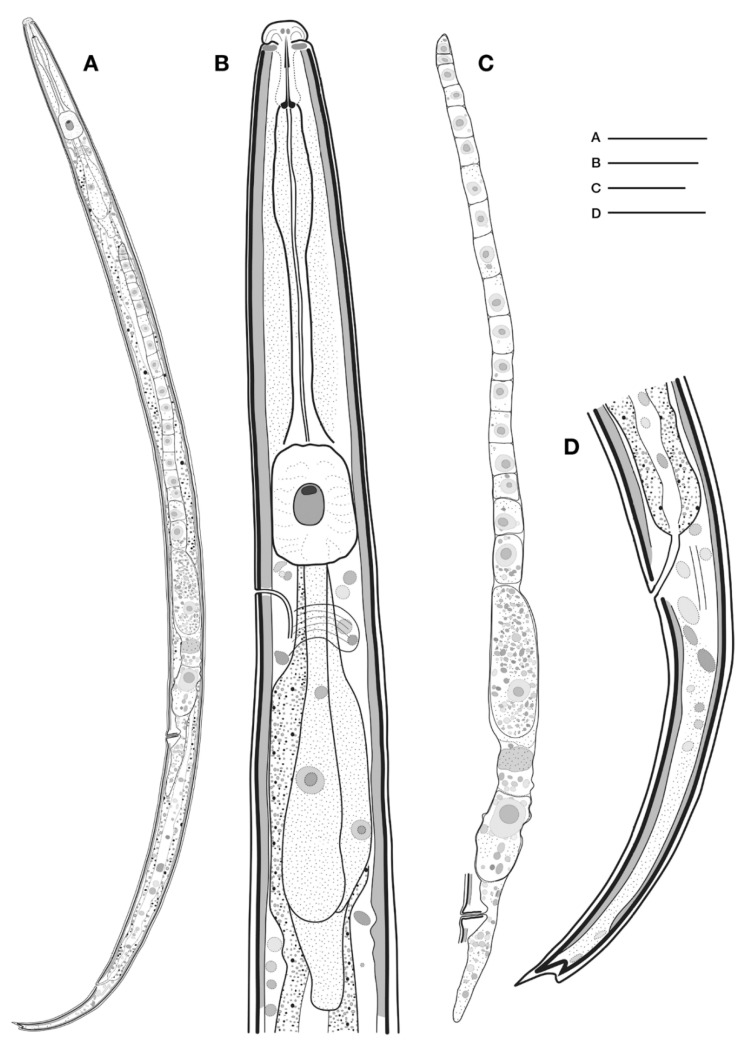
*Aphelenchoides bicaudatus* (Imamura, 1931) Filipjev and Schuurmans Stekhoven, 1941. (**A**) Entire female; (**B**) neck region; (**C**) female reproductive system; (**D**) female posterior region. Scale bars: (**A**) = 50 μm, (**B**,**D**) = 10 μm, (**C**) = 20 μm after Kim et al. [52]. Courtesy of *Animal Systematics Evolution and Diversity Journal*.

Females of *A. bicaudatus* (Figure 4) can be differentiated from other members of the genus by having an unevenly bifurcated tail tip with prongs of different lengths [51].

Male: extremely rare. 

Distribution

*A. bicaudatus* was recorded in most of the tropical and subtropical regions of the world as well as some warmer temperate areas [21]. More specifically, it was reported in the following countries: Australia, Brunei, France, Japan, USA, Russia, Venezuela [28], South Korea [52], Taiwan [32].

## 10. Strawberry Crimp Nematode (*Aphelenchoides fragariae* (Ritzema Bos, 1891) Christie, 1932)

**Figure 5:**
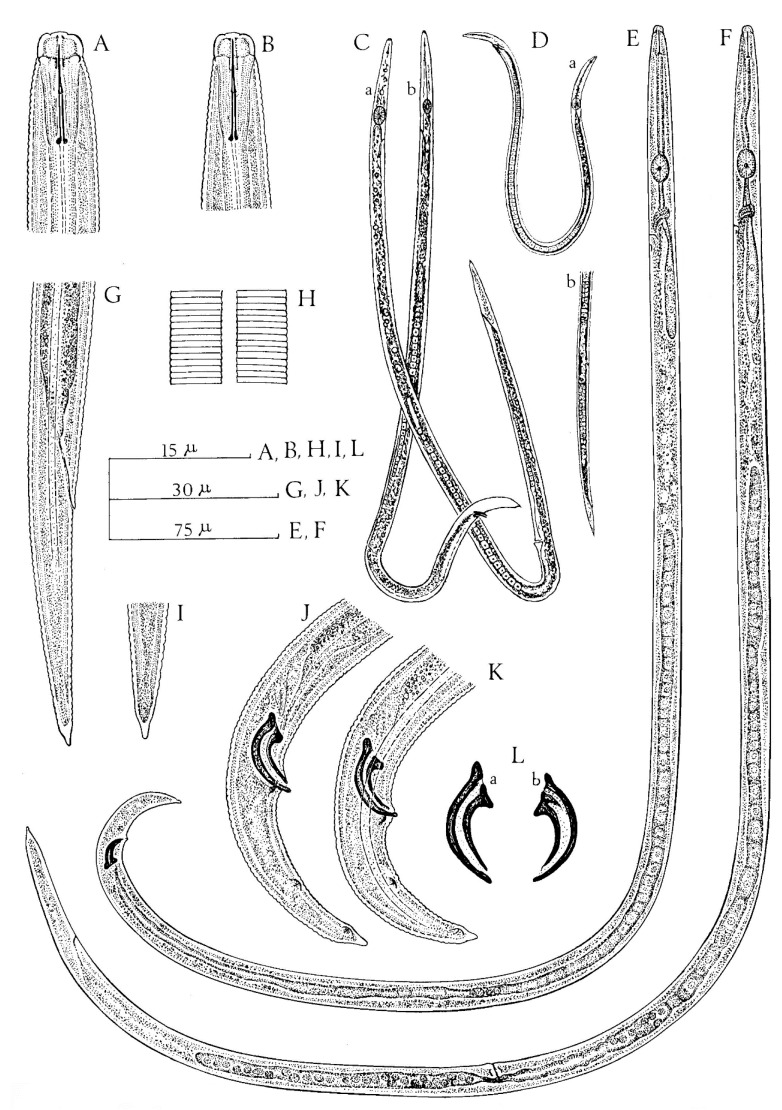
*Aphelenchoides fragariae*. (**A**) Female head end. (**B**) Male head end; (**C**) a, female; b male of *A. olesistus* Ritzema Bos, 1893 (= *A. fragariae*); (**D**) a, male; b, posterior portion of female, of *Aphelenchus fragariae* Ritzema Bos, 1891; (**E**) male; (**F**) female; (**G**) female tail; (**H**) lateral field; (**I**) female tail tip (**J**,**K**) male tails. (**L**) Spicules a, drawn from paratypes of Allen (1952); b, from specimens ex *Cornus canadensis* from Surrey, England after Siddiqi [29]. Courtesy of *Commonwealth Institute of Helminthology*.

*Aphelenchoides fragariae* was originally described by Ritzema Bos (1891) in specimens recovered from strawberry plants sent to him from England (Figure 5). When compared to all the *Aphelenchoides* species mentioned previously, it has the widest distribution as well as hosts range (more than 600 species), to include ferns, herbaceous perennials and bedding plants [2,33,53]. *A. fragariae* is an ecto- and endo-parasite of the above ground parts of a plant, but it can also be mycetophagous [2,21,33]. The nematodes enter the plant leaves through stomata or wounds [1,47]. In the leaves, nematodes feed on mesophyll cells which causes characteristic vein delimited lesions [1,47]. *A. fragariae* survives overwinter in soil, dormant buds, dry leaves, but not in roots [18,47]. Research showed that *A. fragariae* nematodes can tolerate temperature as high as 40 °C and as low as −80 °C once in leaf tissues [18].

Measurements

After Allen [19].

Females: length = 0.45–0.80 mm; a = 45–60; b = 8–15; c = 12–20; V%= 64–71. Males: length = 0.48–0.65 mm; a = 46–63; b = 9–11; c = 16–19; T% = 44–61.

After Franklin [54].

Females: length = 0.552–0.886 (0.796) mm; a = 36–63 (53); body width = 12–17 (15) µm.

Males: length = 0.573–0.864 mm; a = 40–63; body width= 12–17 (14) µm.

After Khan et al. [33].

Females (*n* = 7): length = 0.620–0.895 mm; a = 46.2–64.5; b = 9.0–13.2; c = 13.4–20.3, V% = 66.5–72.2; stylet = 10.0–11.5 µm.

Males (*n* = 7): length = 0.480–0.623 mm; a = 45.7–61.7; b = 9.3–10.8; c = 15.7–18.5, T% = 45.6–60; stylet = 10.0–11.2 µm; spicules = 16.9–19.0 µm.

After Chizhov et al. [34].

Females (*n* = 25): length = 0.525–0.685 (0.579 ± 0.043) mm; a = 37.1–59.8 (48.7 ± 4.8); b = 7.6–9.1 (8.1 ± 0.3); c = 15.2–20.6 (17.0 ± 1.2), c’ = 3.6–5.7 (4.7 ± 0.3); V = 65.0–74.0% (69.0 ± 2.0); stylet = 8.0–11.0 (9.0) µm; head region width = 4.0–5.0 µm; head region high = 3.0 µm; distance from anterior end to: medial bulb base = 52.0–64.0 (58.0) µm, nerve ring = 63.0–78.0 (72.0) µm, excretory pore = 68.0–85.0 (76.0) µm and esophageal gland base = 100.0–150.0 (128.0) µm; post uterine sac length = 58.0–98.0 (77.0) µm; tail length = 28.0–40.0 (34.0) µm; body width at vulva level = 10.0–16.0 (12.0) µm and anus level = 6.0–8.0 (7.0) µm.

Males (*n* = 24): length = 0.435–0.562 (0.493 ± 0.037) mm; a = 41.2–54.8 (46.8 ± 3.1); b = 6.5–8.1 (7.2 ± 0.4); c = 15.9–24.1 (18.5 ± 1.8); stylet = 8.0–10.0 (9.0) µm; head region width = 4.0–5.0 µm; head region height = 3.0 µm; spicule length = 10.0–13.0 (12.0) µm; distance from anterior end to: medial bulb base = 52.0–62.0 (57.0) µm and esophageal gland base = 100.0–135.0 (118.0) µm; nerve ring = 68.0–77.0 (71.0) µm; excretory pore = 70.0–82.0 (76.0) µm; testis length = 204.0–289.0 (250.0) µm; maximal body width = 10.0–13.0 (11.0) µm; tail length = 21.0–33.0 (27.0) µm.

Description

Body very slender (a = 45–63 µm), straight or arcuate when relaxed. Cuticle marked by fine transverse striae about 0.9 µm apart; lateral field with two incisures, 1/7th of body-width. Cephalic region, smooth, anteriorly flattened with straight to curved side margins, almost continuous with neck contour. Lips without annulation. Stylet slender, approximately 10 µm long, with small but distinct basal knob. Median esophageal bulb well developed, oval. Nerve ring about one body width behind median bulb. Excretory pore level at or close behind nerve ring. Esophageal glands stretched five body widths behind the medium bulb, joining esophagus immediately behind the medium bulb. Tail elongate-conoid, terminus bearing a terminal peg which is simple, spike-like.

Female: vulva a transverse slit, at approximately 64–71% of body. Spermatheca elongate-oval. Posterior uterine sac more than half the vulva-anus distance, often containing sperm. Ovary single, with oocytes in a single row. Tail terminus with a single mucronate points point enlarged at the base.

Male: abundant. Male tail curved to about 45–90 degrees. Three pairs of ventro-submedian copulatory papillae (1st slightly post-anal, 2nd midway, and 3rd near the end). Testis single, outstretched; sperm large-sized, rounded, in a row. Spicules large and prominent, ventrally curved, rose-thorn-shaped, with moderately developed dorsal and ventral processes (apex and rostrum) at proximal end; dorsal limb 14–17 µm long.

Distribution

*A. fragariae* has a widespread distribution in Europe, Russia, Japan and North America [21]. According to the CABI Invasive Species Compendium [47], *A. fragariae* is currently reported to be present in 37 countries.

## 11. Chrysanthemum Nematode (*Aphelenchoides ritzemabosi* (Schwartz, 1911) Steiner and Buhrer) 

*Aphelenchoides ritzemabosi* (Schwartz, 1911) Steiner and Buhrer, also known as the Chrysanthemum foliar nematode, is a common plant-parasite infecting more than 300 plant species, second only to *A. fragariae* [2] in the *Aphelenchoides* genus based on the number plants they parasitize. 

Measurements

After Allen [13].

Females: length = 0.77–1.2 mm; a = 40–54; b = 10–13; c = 18–24; V% = 66–75.

Males: length = 0.70–0.93 mm; a = 31–50; b = 10–14; c = 16–30; T% = 35–64.

After Chizhov et al. [29].

Females (*n* = 15): length = 0.768–1.027 (0.916 ± 0.067) mm; a = 43.4–60.5 (51.2 ± 3.7); b = 8.1–9.5 (9.1 ± 0.3); c = 16.8–21.2 (19.3 ± 1.1); c’ = 4.0–5.1 (4.6 ± 0.2); V% = 68–71 (69 ± 0.2); stylet = 9.0–11.0 (10.0) µm; head region width = 6.0–7.0 µm; head region height = 3.0 µm; distance from anterior end to: medial bulb base = 71.0–77.0 (74.0) µm; nerve ring= 95.0–108.0 (100.0) µm; excretory pore= 108.0–130.0 (121.0) µm and esophageal gland base = 145–185 (170) µm; postuterine sac length= 105.0–160.0 (134) µm; tail length= 41.0–54.0 (48.0) µm; body width at vulva level = 16.0–23.0 (18.0) µm and anus level= 8.0–12.0 (10.0) µm.

Males (*n* = 15): length = 0.625–0.852 (0.721 ± 0.053) mm; a = 36.9–53.3 (46.3 ± 3.3); b = 6.5–9.4 (7.9 ± 0.6); c = 17.3–22.4 (19.9 ± 1.1); stylet = 9.0–11.0 (10.0) µm; head region width = 6.0–7.0 µm; head region height= 3.0 µm; spicule = 15–18 (16) µm; distance from anterior end to: medial bulb base = 67.0–72.0 (69.0) µm, nerve ring = 85.0–108.0 (93.0) µm; excretory pore = 92.0–118.0 (105.0) µm and esophageal gland base = 156.0–180.0 (169.0) µm; testis length = 353.0–512.0 (442.0) µm; tail length = 34.0–39.0 (36.0) µm.

**Figure 6:**
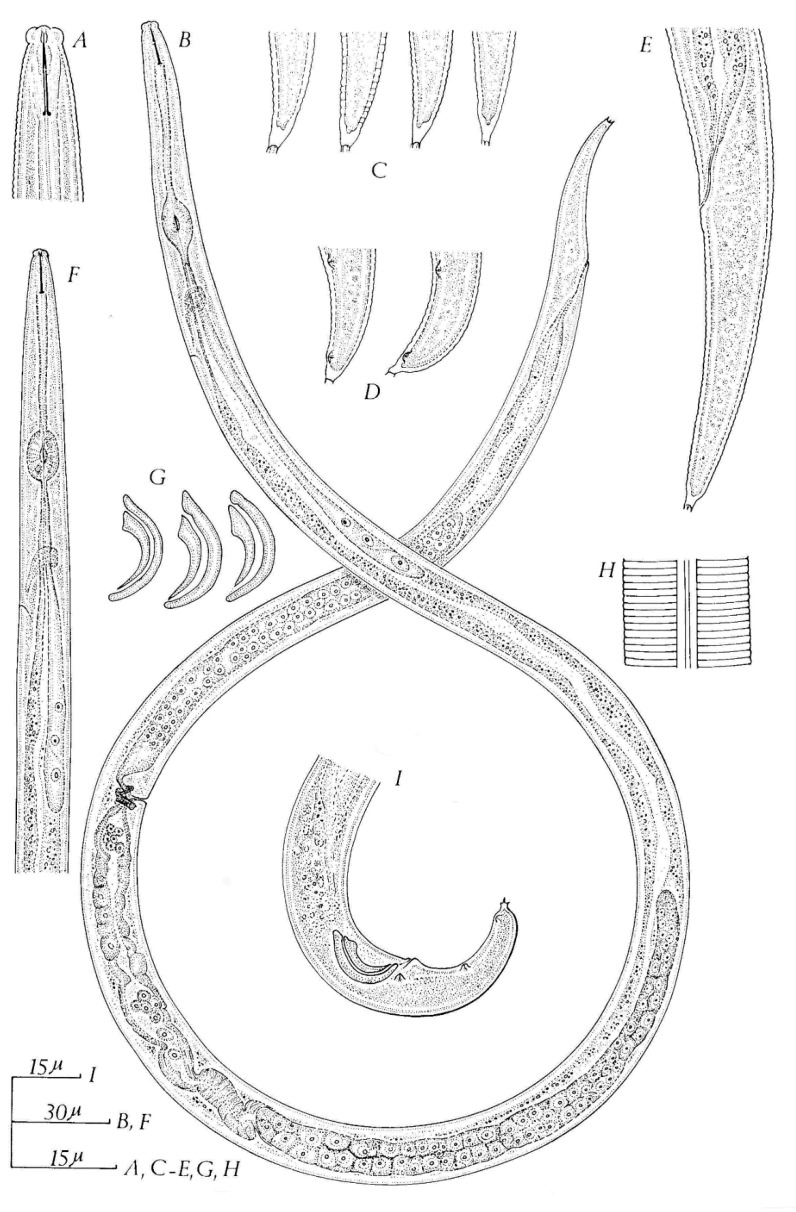
*Aphelenchoides ritzemabosi.* (**A**) Female head; (**B**) female; (**C**) female tail ends; (**D**) male tail ends; (**E**) female tail; (**G**) spicules; (**H**) lateral field; (**I**) male tail region. (**A**, **E**, and **F** syntypes; **B**, **C**, and **H** Specimens from chrysanthemum, Stockholm; I Specimen from *Buddleia* leaf, Sussex, England) after Siddiqi [25]. Courtesy of *Commonwealth Institute of* *Helminthology*.

Description (Figure 6)

Female: nematodes with slender body, with fine transverse striae on the cuticle. Four lines present in the lateral field. Lip region set off, wider than neck at base of lips with no annulations. Hexaradiate framework weakly sclerotized. Stylet approximately 12 µm long, with small but well-developed basal knobs. Median esophageal bulb well developed, oval in shape. Nerve ring 1.5 body widths behind median bulb. Excretory pore located behind nerve ring, approximately 0.5–2 body widths posterior to nerve ring. Esophageal glands extending 4 body widths over the intestine, joining esophagus immediately behind median bulb. Oocytes in multiple rows, several in a cross-section at middle of ovary. Posterior uterine branch extending for more than half the vulva-anus distance, usually containing sperms. Tail elongated-conoid. Terminus peg-like armed with two-four small mucronate points pointing posteriorly. 

Male: males are common, having a tail curvature at about 180 degrees when relaxed. Testis single. Three pairs of ventro-submedian papillae. First pair adanal, second midway on tail, third near end. Spicules smoothly ventrally curved, the ventral piece without a ventral process at the distal end; dorsal limb 20–22 µm long. Terminus peg-like armed with two-four small mucronate points.

Distribution

*Aphelenchoides ritzemabosi* is a major pest of chrysanthemum in Europe, Russia, North America, South Africa, New Zealand, Australia, and Brazil [25]. According to the CABI Invasive Species Compendium [55], *A. ritzemabosi* is currently reported to be present in 35 countries around the world. 

Identification

Accurate identification of foliar nematodes (*Aphelenchoides* spp.) is crucial for effective disease control. Major efforts should be geared towards rapid and accurate classification of the pathogens so that appropriate control measures could be taken. In addition, timely and accurate diagnosis is also needed to make sound decisions regarding quarantine of imported and exported plant material and commodities. Nevertheless, the identification of foliar nematodes to species level remains a challenging endeavor. The diagnosis and/or relationship between conserved morphology, variable morphometrics, host effects, intraspecific variation, existence of cryptic species, and the ever-increasing number of described species, still vary significantly. To add to the confusion, there is verification of mixed populations and/or detection of rare species which require(s) identification techniques, including morphology of adult females; male, and labial region shape, and stylet morphology; V% age, body length, and shape of tail and tail terminus, and, in some cases, biochemical or molecular methodologies. Because of an increasing number of described species, the value of many of these characters often show large intraspecific variation. Isozyme electrophoresis has discriminated a number of these otherwise cryptic species. Currently used PCR-based molecular methodologies offer hope for a future relying on bigger genebanks that could be used by scientists for a more accurate specie identification. Integrated morphology and molecular approaches are essential to future improved identification of Anguinata nematodes. Detailed diagnostic characters differentiating various species of foliar nematodes have been given by authors such as Allen [19], Hunt [21], Shahina [22]. 

## 12. Genus *Litylenchus* Zhao, Davies, Alexander and Riley, 2011

Genus *Litylenchus* Zhao, Davies, Alexander and Riley, 2011 is a new genus with much smaller number of species when compared to *Aphelenchoides* genus. *Litylenchus crenatae* Kanzaki, 2019*, Litylenchus crenatae mccannii* Carta 2020, are emerging foliar pathogens of major economic importance. Nematodes from this genus parasitize trees (*Fagus grandifolia*) and bushes (*Coprosma repens*). *Litylenchus crenatae mccannii* described by Carta et al. [11] seems to be a very aggressive subspecies with devastating effects on beech trees (*Fagus grandifolia*). Even though *Litylenchus crenatae mccannii* was initially found infesting beech trees in Ohio [11], it was also reported in several other states and provinces, to include Pennsylvania, New York, Ontario, Canada [56], Connecticut [57], New Jersey, Rhode Island, and West Virginia (unpublished data).

After Zhao [35] the genus *Litylenchus* Zhao, Davies, Alexander and Riley, 2011 is characterized as follows:

- Adults and juveniles of *Litylenchus* gen. from within leaves not forming galls;
- Lacking obese females with a spiral form;
- Slender to semi-obese, cylindrical nematodes, barely curved around ventral axis;
- Lack of sexual dimorphism in head, pharyngeal, and tail characters;
- Cuticle with fine annulations, head offset;
- Stylet short (9–12 μm), robust, with rounded knobs;
- Pharynx with non-muscular fusiform median bulb, valve may be present;
- Pharyngeal glands contained in a large terminal bulb abutting intestine and three large nuclei present;
- Secretory/excretory pore opening 1–1.5 body diameter posterior to nerve ring;
- Female with mono-prodelphic gonad with quadricolumella and post-uterine sac;
- Male with arcuate spicules and simple gubernaculum;
- Bursa arising 1–2 cloacal body diameter anterior to cloacal aperture, extending nearly to tail tiptail medium, conoid, tip shape variable, usually bluntly rounded in male, more variable in female.

## 13. Systematic Position

Based on phylogenetic analyses, *Litylenchus* genus [35] is close to *Subanguina.* However, the two genera have many morphological differences as highlighted below:

- *Litylenchus* genus. does not induce typical galls like *Anguina* and *Nothanguina*;
- Lack of obese females with a spiral form in *Anguina* and *Nothanguina* and lack of semi-obese females in *Ditylenchus*;
- Stylet of *Litylenchus* genus is more robust and the stylet knobs are rounded compared to *Ditylenchus*;
- Excretory pore situated posterior to nerve ring;
- Tails of *Litylenchus* genus are conoid rather than elongate conoid to filiform in *Ditylenchus,* and elongate conoid in *Nothotylenchus* gen.;
- Males have a shorter bursa compared to those of *Nothotylenchus* gen.

List of *Litylenchus* species and synonyms:

Type species:

1. Litylenchus coprosma

Other species

1. *Litylenchus crenatae*
2. *Litylenchus crenatae mccannii*

## 14. *Litylenchus coprosma* Zhao, Davies, Alexander and Riley, 2011 

Measurements

After Zhao et al. [35].

Slender female (*n* = 13): L = 743 ± 50 (649–816) µm; a = 55.2 ± 4.0 (51.5–63.3); b = 4.4 ± 0.6 (3.9–5.8); c = 18.7 ± 1.3 (16.3–21.3); V %= 81.5 ± 2.4 (76.5–85.3); stylet = 10.8 ± 0.9 (8.9–11.7) µm.

Obese female (*n* = 15): L = 856 ± 72 (710–940) µm; a = 32.8 ± 3.7 (24.9–37.7); b = 5.1 ± 0.6 (4.2–6.8); c = 19.4 ± 2.5 (15.4–25.0); V%= 82.2 ± 1.6 (78.8–84.7); stylet = 10.9 ± 0.3 (10.2–11.4) µm.

Male (*n* = 11): L = 899 ± 66 (768–994) µm; a = 52.0 ± 4.4 (44.5–60.2); b = 5.4 ± 0.4 (4.8–6.2); c = 21.1 ± 1.9 (18.2–24.1); stylet = 10.5 ± 0.5 (9.7–11.3) µm; spicule= 16.2 ± 0.7 (14.9–17.0) µm.

Description

**Figure 7:**
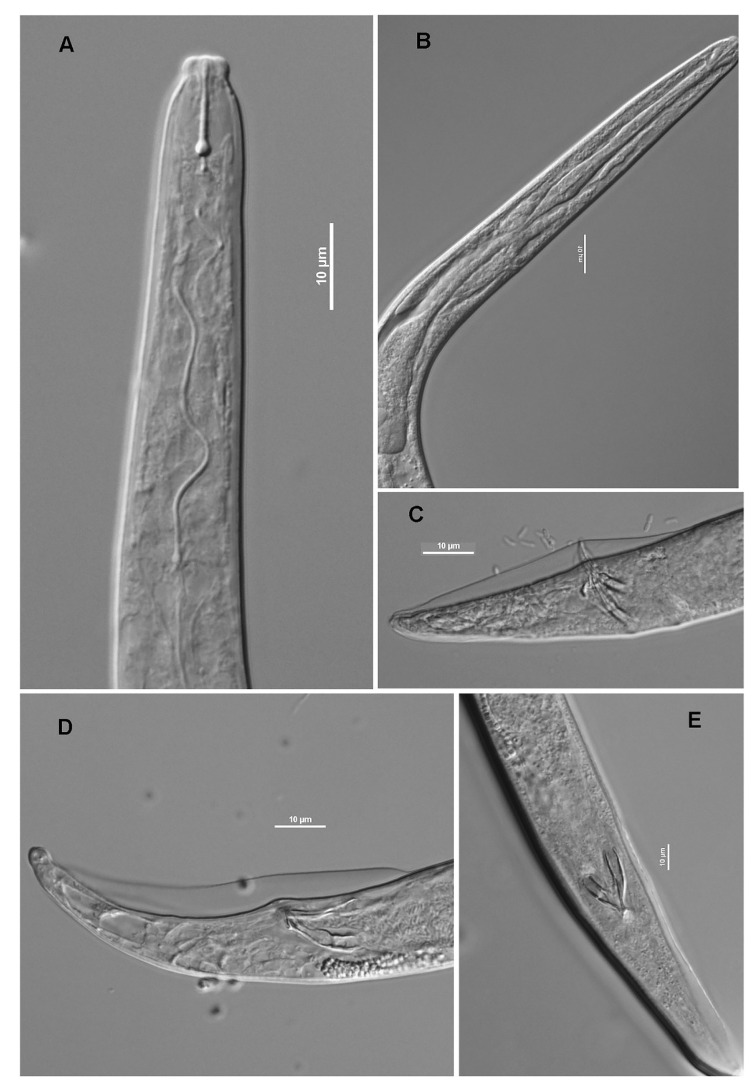
*Litylenchus coprosma*. All males in lateral view, except E which is ventral. (**A**) Anterior region; (**B**) pharynx showing median bulb; (**C**) tail with bursa; (**D**) tail showing spicules and variation in shape of tail tip; (**E**) spicules. (Scale bars = 10 μm) after Zhao et al. [35]. Courtesy of *Nematology*.

**Figure 8:**
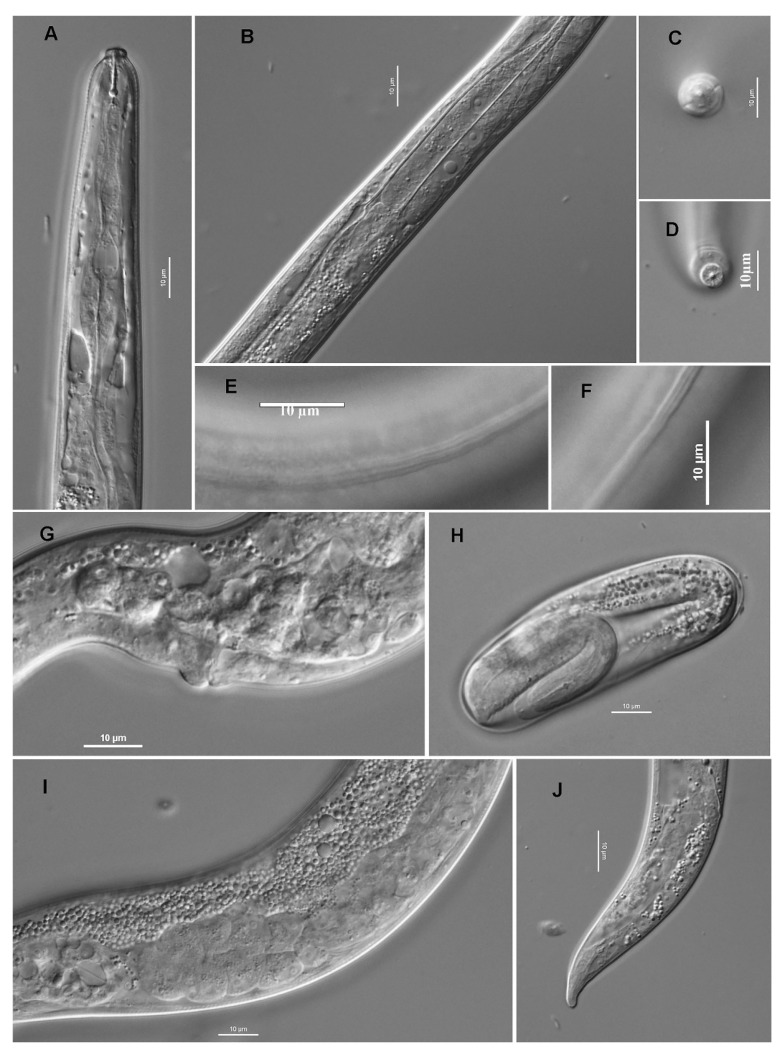
*Litylenchus coprosma*. All in lateral view, except C, D en face. (**A**) Head of mature, semi-obese female; (**B**) terminal pharyngeal bulb; (**C**) sub-terminal head showing amphidial apertures; (**D**) apical view of head; (**E**) lateral fields at mid-body showing four incisures; (**F**) lateral fields at pharyngeal region showing three incisures; (**G**) vulva and post-uterine sac; (**H**) second-stage juvenile within egg; (**I**) quadricolumella; (**J**) female tail. (Scale bars = 10 μm) after Zhao et al. [35]. Courtesy of *Nematology*.

*Litylenchus coprosma* has adult females with two distinct forms, one described as semi-obese (a = 20–40) and the other slender (a = 45–65) (Figure 7 and Figure 8).

Semi-obese female: when killed by heat body is almost straight, semi-obese. Maximum body width is at mid-body. Body cuticle finely striated, almost smooth. Four lines can be observed in lateral field extending almost to tail terminus. Head offset, cephalic framework, and stylet as described for male. Excretory pore located ca 3–3.5 body diameter from anterior, opening near anterior end of terminal bulb, duct with obvious cuticular lining. Hemizonid, pharynx, pharyngeal glands, and pharyngo-intestinal junction as described for male. Nerve ring is located approximately 100 μm from anterior extremity. Deirids and phasmids not seen. Gonads are monodelphic, prodelphic, outstretched, crustaformeria forming a quadricolumella. Oocytes arranged in single row. Oviduct with several cells forming a valve just anterior to elongate, sac-like spermatheca. Vulva located 7–11 anal body diameter anterior to anus (80–85% of body length). Vulval slit occupying almost half body diameter when viewed laterally, vagina almost perpendicular to body wall. Post-uterine sac extending 20–70% of distance from vulva to anus, approximately 2.7 anal body long, sometimes with sperms, lacking cellular relicts of posterior ovary. Rectum difficult to see, anus pore-like, opening in a cuticular depression. Tail approximately 4–5 anal body diameter long, conoid, straight, with a variable tail terminus, may be bluntly rounded, more or less bifurcate, or appear bilobed. Mucro not observed.

Slender female: very similar to the semi-obese females, but slender. Head capsule is a little bit bigger, 59–77% of body diameter at level of stylet knobs compared to the semi-obese females, where the head capsule is between 48–62%. Quadricolumella cells are smaller than in semi-obese female.

Male: when killed, the nematodes assume a smoothly ventrally arcuate shape, body cylindrical, narrowing to a bluntly rounded conoid tail. Body cuticle smooth with three incisures in the lateral field visible in the region of procorpus increasing to four incisures at mid-body and extending almost to tail tip. Head is set off from the body, smooth, and not annulated. Lightly sclerotized cephalic framework with six sectors.

En-face view shows amphidial apertures appearing as small lateral slits. Stylet robust, with well-developed rounded knobs, conus comprising ca 40% of stylet length, diameter narrowing sharply to be distinctly less than that of shaft. The opening of dorsal esophageal gland is located just posterior to stylet knobs. Nerve ring is located 70–110 μm from anterior extremity, surrounding isthmus, *ca* one body diameter long. Excretory pore is located ca 5–6 body diameter from anterior end, opening posterior to nerve ring. Hemizonid located immediately anterior to excretory pore. Procorpus cylindrical, fusiform, non-muscular median bulb which is approximately one body diameter long and narrowing sharply to isthmus which is slender, cylindroid, marked off from terminal bulb, pharyngeal glands enclosed in a pyriform terminal bulb containing three large nuclei. Esophago-intestinal junction is immediately posterior to terminal bulb and covered by it in some specimens, valve present, without hyaline cells. Deirids and phasmids were not observed. Testis outstretched, reflexed in some specimens, reaching to nerve ring in some specimens, with spermatocytes arranged in a single row. Spicule paired, similar, arcuate, 2–3 μm wide at anterior end, gradually narrowing towards tip. Capitulum absent. Gubernaculum simple and arcuate. Tail conoid with a variable in shape tail terminus, usually bluntly rounded, but may have terminal process; no mucron observed. Bursa membranous, crenate in some, arising ca 1–2 cloacal body diameter anterior to cloacal aperture, extending nearly (90–95% of tail length) to tail tip. 

Distribution

*Litylenchus coprosma* was reported in New Zealand from *Coprosma repens* [35] and from *Coprosma robusta* [26].

## 15. *Litylenchus crenatae* Kanzaki, Ichihara, Aikawa, Ekino, and Masuya, 2019

Measurements

After Kanzaki et al. [37].

Mature female (*n* = 10): L = 816 ± 32 (758–870) µm; a = 35.9 ± 3.4 (30.2–41.1); b = 6.6 ± 0.4 (6.1–7.6); c = 24.5 ± 1.9 (21.8–28.1); V%= 81.5 ± 1.0 (79.4–83.2); stylet = 10.6 ± 0.5 (9.9–11.3) µm.

Immature female (*n* = 10): L = 868 ± 33 (837–915) µm; a = 67.5 ± 5.8 (60.7–74.4); b = 4.3 ± 0.3 (3.9–4.8); c = 15.7 ± 0.7 (14.4–16.7); V% = 77.4 ± 0.5 (76.6–78.3); stylet = 8.0 ± 0.4 (7.4–8.5) µm.

Mature male (*n* = 9): L = 805 ± 21 (766–840) µm; a = 41.0 ± 2.4 (37.4–44.4); b = 6.4 ± 0.4 (5.9–7.3); c = 24.8 ± 2.5 (21.4–30.3); stylet = 10.5 ± 0.4 (9.9–11.3) µm; spicule = 18.3 ± 1.0 (16.7–20.2) µm; gubernaculum = 8 ± 0.4 (7.1–8.5) µm.

Immature male (*n* = 8): L = 707 ± 41 (642–773) µm; a = 57.2 ± 4.7 (48.9–61.9); b = 5.3 ± 0.6 (4.5–6.3); c = 21.1 ± 2.0 (18.5–25.1); stylet = 10.2 ± 0.4 (9.9–11.0) µm; spicule = 15.6 ± 1.2 (14.2–17.7) µm; gubernaculum = 6.5 ± 0.4 (6.0–7.1) µm.

Description

**Figure 9:**
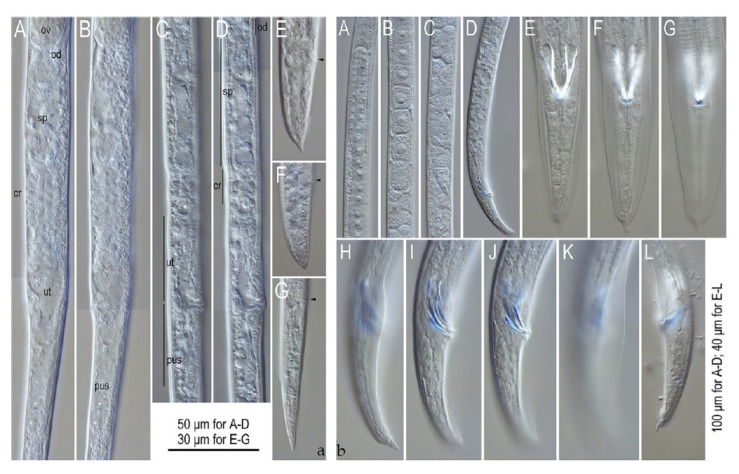
Males and Females of *Litylenchus crenatae.* (**a**) Female reproductive system and tail of *Litylenchus crenatae;* (**A**,**B**) posterior part of gonad of mature female in different focal planes; (**C**,**D**) posterior part of gonad of immature female in different focal planes; (**E**,**F**) tail of mature female; (**G**) tail of immature female. Ovary (ov), oviduct (od), spermatheca (sp), crustaformeria (cr), uterus (ut), and post-uterine sac (pus) are shown in (A–D), and anal opening is indicated by arrowheads in (**E**–**G**). (**b**) Male reproductive system of *Litylenchus crenatae* (**A**–**K**) are mature individuals, (**L**) is an immature individual. (**A**) Anterior end of testis; (**B**) middle part of mature testis; (**C**) posterior part of testis; (**D**) posterior end of testis and *vas deferens;* (**E**,**G**) ventral view of tail in different focal planes; (**H**,**K**) right lateral view of tail in different focal planes; (**L**) left lateral view of tail of immature individual. After Kanzaki et al. [32]. Courtesy of Nematology.

Female (Figure 9a): when killed, the nematodes assume a smoothly ventrally arcuate shape, body cylindrical, vermiform to semi-obese. Anterior part and cuticular morphology similar to mature male. Female gonad single, anteriorly outstretched reaching to level of pharyngeal glands. Oocytes are arranged in single row in entire ovary. Oviduct is short and spermatheca is elongated oval filled with large sperm, posteriorly connected to crustaformeria, which consists of four rows of four large and rounded cells, i.e., forming a quadricolumella, posteriorly connected to uterus by a cluster of small cells. Uterus, a thick-walled tube, sometimes containing an egg. Vagina at right angles to body axis or slightly inclined anteriorly. Vulva, a horizontal slit. Post uterine sac present, well-developed, with a thin wall and a short appendage comprising several rounded cells at distal end. Rectum is about less than one anal body diameter in length, with muscular constriction at intestine-rectal junction. Tail is short and broad, abruptly narrowing at the end with a conoid and bluntly pointed terminus, sometimes appearing like a conical blunt mucron.

Male (Figure 9b): when killed, the nematodes assume a smoothly ventrally arcuate shape, body cylindrical, not clearly obese or semi-obese. Body cuticle annulated with six incisures in the lateral field at the anterior part of body, 6–8 incisures around mid-body, and posteriorly connected to bursa. Deirids present in middle of lateral field slightly posterior to hemizonid and excretory pore. Lip region slightly offset from body, with a truncated shape, separated by a very shallow constriction.

**Figure 10:**
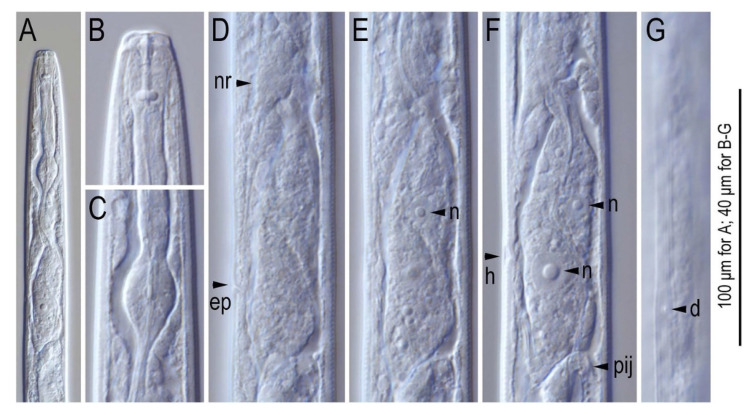
Anterior region of mature adults of *Litylenchus crenatae*; (**A**) anterior end to pharyngo-intestinal junction; (**B**) lip region; (**C**) metacorpus (median bulb); (**D**–**G**) pharyngeal gland region in different focal planes. Nerve ring (nr), excretory pore (ep), pharyngeal gland nuclei (n), hemizonid (h), pharyngo-intestinal junction (pij) and deirid (d) are indicated in (**D**–**G**) after Kanzaki et al. [37]. Courtesy of *Nematology*.

Stylet with narrow lumen and a shaft with prominent rounded basal knobs (3.6 μm in diameter). Dorsal esophageal gland is located posterior to stylet knobs. Procorpus is cylindrical. Median esophageal bulb is weakly developed, with small metacarpal valve at mid-bulb length. Isthmus is cylindrical, but narrower than the procorpus, enveloped by the nerve ring in its mid-length. Broad and glandular gland lobe with three large nuclei were observed (Figure 10). Hemizonid found at level the beginning of expansion of pharynx. Excretory pore located slightly posterior to hemizonid, with clear secretory-excretory duct. Nuclei of the esophageal overlap observed between hemizonid and pharyngo-intestinal junction, two being just anterior to the third, and latter located slightly anterior to junction. Gonad single, anteriorly outstretched reaching to level of pharyngeal glands. Testis outstretched with spermatocytes arranged in single row from anterior to middle part of testis and in multiple rows in posterior section. *Vas deferens* is visible, consisting of rounded cells, sometimes containing well-developed sperm. Spicules paired, smoothly arcuate ventrally, forming a smoothly curved horn-like blade with bluntly pointed distal end in lateral view (V-shaped). Gubernaculum simple, crescent or bow-shaped in lateral view. Bursa peloderan, well developed arising three cloacal body diameter anterior to cloacal opening and terminating near tail tip. Tail is conoid, bluntly pointed in lateral view. 

Distribution

*Litylenchus crenatae* was reported so far from Japan from *Fagus crenata* [37].

The phylogenetic relationships among anguinid nematodes inferred from three ribosomal RNA loci were provided by Kanzaki et al. [37]. The marker sequences derived from *Litylenchus crenatae* specimens, LC383723 (SSU), LC383725 (D2-D3 LSU), and LC383724 (ITS) were deposited to GenBank. 

## 16. *Litylenchus crenatae* Kanzaki et al., 2019 *mccannii* ssp. Carta, Handoo, Li, Kantor, Bauchan, McCann, Gabriel, Yu, Reed, Koch, Martin, Burke 2020

Measurements

After Carta et al. [11].

Immature female (*n* = 10): L = 823 ± 61 (750–947) µm; a = 72.9 ± 3. (61.0–86.0); b = 5.4 ± 0.7 (4.5–6.6); c =17.4 ± 3.3 (13.0–25.0); V%= 76.9 ± 1.2 (75.0–79.0); stylet = 9.7 ± 0.9 (8.5–11.2) µm.

Mature male (*n* = 4): L = 548 ±16.7 (534.5–566.7) µm; a = 36.1 ± 5.4 (33.4–44.1); b = 4.8 ± 0.2 (4.6–4.9); c = 15.5 ± 0.2 (15.3–15.9); stylet = 11.1 ± 0.5 (10.5–11.4) µm; spicule= 16.3 ± 1.4 (14.9–17.6) µm; gubernaculum = 5.3 ± 0.8 (4.3–6.1) µm.

Description

**Figure 11:**
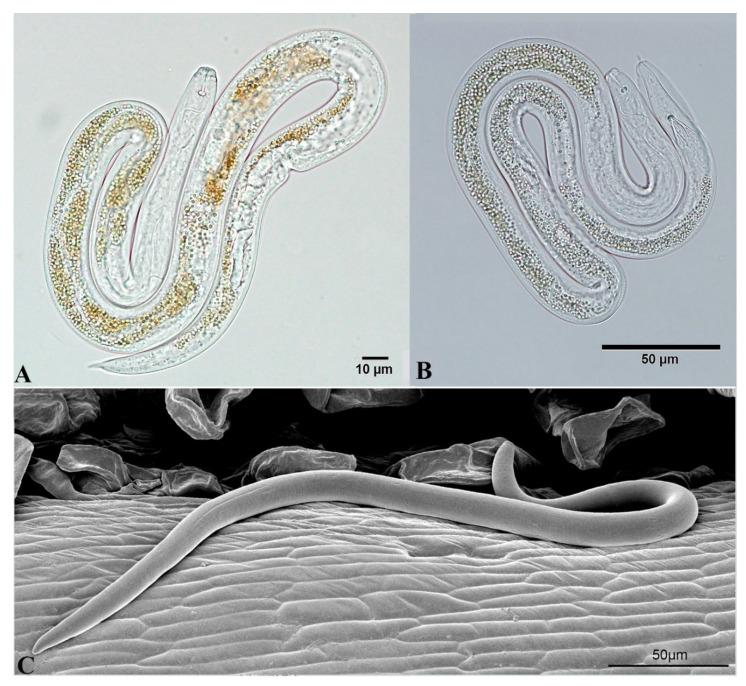
Males and Females of *Litylenchus crenatae mccannii*. (**A**) Mature Female; (**B**) male; (**C**) LT-SEM of young Female. Courtesy of Gary Bauchan and Shiguang Li of Electron and Confocal Microscopy and Mycology and Nematology Genetic Diversity and Biology Laboratory (MNGDBL), USDA, ARS, Beltsville, MD, respectively.

Females have long and slender bodies, a lip region slightly offset with 5 annules. Stylet measures 9.7 ± 0.9 µm in young females with 5% of the pharynx length, and 7–10% of the pharynx length in males. Median bulb is weak without an obvious valve. The vulval region is kinked and irregular and the anterior gonad is relatively long, nearly five times the length of the post uterine sac. The post uterine sac is about three times the vulval body width and one quarter of the vulval anal distance. The rectum is approximately one quarter of the tail length and the anus is pore-like and obscure in most specimens. Tail is conical, slender and asymmetrically pointed, with a gradually tapering and the tail tip often with mucronate extension (Figure 11). There is a shape variation in tails of immature and mature females.

Female: *Litylenchus crenatae mccannii* ssp. n. young female population from North America can be differentiated from the *Litylenchus crenatae* described from Japan by:

- Having longer stylet 9.7 ± 0.9 µm (8.6–11.2) vs. 8.0 ± 0.4 (7.4–8.5) and longer stylet conus 4.6 µm (3.6–5.2) vs. 3.1 ± 0.2 (2.8–3.5);
- The post- uterine sac in mature females was shorter (36.9 ± 9.4 vs. 68 ± 7.4);
- Tail was shorter in the fixed immature female populations (48.3 ± 6.2 vs. 55 ± 3.8) but it was longer in the mature populations (43.7 ± 11.3 vs 33 ± 2.3) which was also reflected in different c (16.8 ± 1.4 vs 24.5 ± 1.9) and c’ (5.3 ± 1.2 vs. 2.9 ± 0.3) ratios;
- The body width in mature females was narrower in all populations (16.2 ± 2.4 vs. 22.9 ± 2.6).

Male: males of *Litylenchus crenatae mccannii* ssp. n. are very similar to *Litylenchus crenatae* males described from Japan. Carta et al. [11] noted some differences between the North America and the Japan population such as:

- Longer stylet (11.2 (10.6–12) vs. 10.2 (9.9–11)) μm and stylet conus (4.8 (4.4–5.3) vs. 3.6 (3.5–4.3)) μm;
- A wider body (16.7 (13.5–20.3)) μm than the fixed type population from Japan.

Molecularly, *Litylenchus crenatae mccannii* from Ohio, Pennsylvania, and the neighboring province of Ontario, Canada, showed some differences in morphometric averages among females when compared to the Japanese population described by Kanzaki et al. [32]. Ribosomal DNA marker sequences were nearly identical to the population from Japan [11]. The 18S rDNA and internal transcribed spacer (ITS) rDNA sequences for *Litylenchus crenatae* from Japan are 99.9% and 99.7% similar, respectively, to *Litylenchus crenatae mccannii* from North America. A sequence for the COI marker was also generated, although it was not available in the Japanese population [11]. The marker sequences derived from *Litylenchus crenatae mccannii* specimens, 104H78 and 104H82 were deposited to GenBank with accession numbers for rDNA (MK292137, MK292138) and COI (MN524968, and MN524969). 

**Figure 12:**
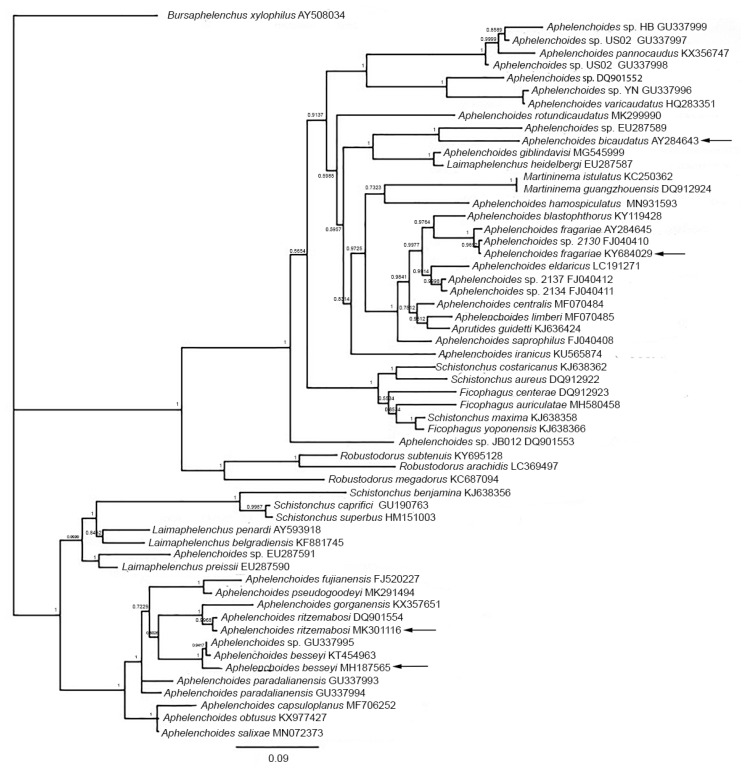
Phylogenetic Bayesian tree of 18S rDNA sequences for *Aphelenchoides* and related genera from multiple sequence alignment made with Clustal Omega (EMBL-EBI, https://www.ebi.ac.uk/Tools/msa/clustalo/); tree processed from 1,100,000 iterations in MrBayes version 3.2.6 [58] within Geneious Prime Version 2020.2.4 (Biomatters, Ltd., Auckland, NZ). Pathogenic species are indicated by arrows.

**Figure 13:**
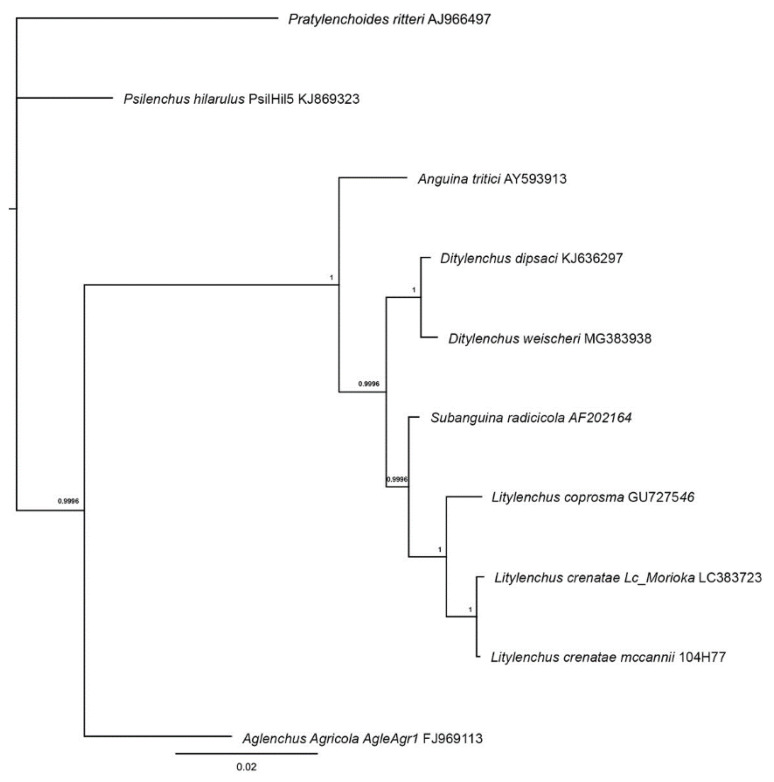
Phylogenetic Bayesian tree from 1,100,000 iterations created in MrBayes version 3.2.6 [59] from multiple sequence alignment made with Clustal Omega (EMBL-EBI, https://www.ebi.ac.uk/Tools/msa/clustalo/) within Geneious Prime Version 2020.2.4 (Biomatters, Ltd., Auckland, NZ).

Phylogenetic trees for 18S rDNA of *Aphelenchoides* and *Litylenchus* are shown in Figure 12 and Figure 13.

## 17. Conclusions and Future Prospects

Until recently, morphology used to be the only way to differentiate nematodes. With recent developments of molecular approaches in taxonomy gaining more widespread use, molecular identification has the potential to become an indispensable tool in the near future. As the GenBank continues to expand, molecular identification can become a reliable resource for nematode identification. Classical morphology continues to play a very important role in nematode identification, being reliable, cheap and quick. Molecular approaches can complement classical morphology and are crucial for species with similar morphological characters. A blend of both morphological (including SEM), morphometric, and molecular data is essential for future new foliar nematode species. The prospects in foliar nematode taxonomy and diagnostics are dependent on molecular-based methodologies that will discriminate not only species but also at the level of host races and pathotypes. This finer discrimination provides opportunities for more focused management strategies. These techniques can provide rapid diagnostics and help resolve the present problems associated with morphologically conservative organisms. When widely employed, these characterization techniques will allow differentiation between nominal species, also enhancing our understanding of the phylogeny of the genus and its relationship with other plant-parasitic nematodes.
